# AAV-iRFP labelling of human mesenchymal stem cells for near-infrared fluorescence imaging

**DOI:** 10.1042/BSR20160556

**Published:** 2017-04-28

**Authors:** Can Huang, Wenjun Lan, Feifei Wang, Chun Zhang, Xiaomei Liu, Qin Chen

**Affiliations:** 1 Shanghai Key Laboratory of Bio-Energy Crops,School of Life Science, Shanghai University, Shanghai 200436, PR China; 2 KAS key Lab of Bio-Medical Diagnostics, Suzhou Institute of Biomedical Engineering and Technology, Chinese Academy of Sciences, Suzhou, Jiangsu 215163, PR China; 3 College of Life Science and Technology, China Pharmaceutical University, Nanjing, Jiangsu 210009, PR China

**Keywords:** Adeno-associated virus, iRFP, in vivo imaging, MSC, Site-specific integration

## Abstract

Near-IR fluorescence (NIRF) imaging is a new technology using IR fluorescent protein (iRFP) gene labelling and is potentially useful for *in vivo* applications. In the present study, we expressed iRFP and the TNF-related apoptosis inducing ligand gene in mesenchymal stem cells (MSCs) using adeno-associated virus (AAV) and showed that iRFP-labelled MSCs can be detected by fluorescence microscopy. We injected mice with MSCs labelled with AAV-iRFP, which we were then able to detect by whole-animal NIRF imaging. Our technique provides a visualizable, convenient and sensitive platform for research on tracking the fate of transplanted MSC cells *in vivo*.

## Introduction

Human mesenchymal stem cells (MSCs) are multipotent cells that can self-renew, proliferate and differentiate into various cell types. They can overcome difficulties associated with immune rejection of transplanted cells and can easily be isolated from the body, cultured *in vitro* and transplanted into patients autologously. In addition, they have a high metabolic activity and robustly express transgenes. Current treatments for most metastatic cancers often have poor outcomes because of the dispersive nature of the disease, toxicity and the inaccessibility of some tumour sites [1–3]. The bottleneck here is that the therapeutic vectors often lead to a variety of innate and adaptive immune responses. Recently, MSCs have been proved to be a potential delivery vector for immune privilege, and these cells may be transduced to high levels with adenovirus and lentivirus, among others. The properties of MSCs may have an important role in protecting cells from immune responses against the transgene that they are carrying [4]. These cells can also regulate the immune response of T cells to novel antigens, thereby decreasing the probability of a cytotoxic T-cell response to the transduced cells through secreting cytokines [5]. In order to use MSCs as transgene delivery vehicles, a high percentage of the cells need to become infected in order to express high levels of transgene and this process must be safe.

Adeno-associated virus (AAV) is a small ssDNA virus that can carry foreign genes and is able to integrate into a specific site on human chromosome 19 (19q13.4) [6], known as AAVS1 and acknowledged around the world as a safe locus. While traditional transfection strategies typically involve the random integration of foreign genes into chromosomes, AAVs have many advantages, including high efficiency of transduction of post-mitotic tissues *in vivo*, long-term stable transgene expression and highly safe without inflammation or an immune response [7]. Consequently, they have been widely applied to the establishment of transgenic cell lines and gene therapy.

Fluorescence of genetically encoded markers within cells is an effective tool to measure many aspects of cell behaviour, including cell proliferation *in vitro* and *in vivo* [8–9].Traditional fluorescent proteins (FPs) like GFP are useful in single-cell research but are undesirable for animal studies due to the high autofluorescence background and limited excitation light penetrance and emission collection efficiency due to the shorter wavelengths used. Luciferase also has its own drawbacks: assays using it work best with cell lysates, making time-course analyses extremely cumbersome and adding variability *in vitro*. In addition, non-destructive luciferase assays depend on cellular ATP and the diffusion of exogenous luciferin, which make absolute quantification of experiments difficult [10].

IR fluorescent protein (iRFP) was originally isolated from the phytochrome of the bacteria *Rhodopseudomonas palustris* [11,12]. IRFP682, with a high penetration depth in tissues, was obtained by genetic engineering of the chromophore-binding domain of phytochrome RpBphP2, afforded variants with further improved fluorescence properties [13]. IRFP682 can brightly label live mammalian cells with emission and excitation spectra at near-IR wavelengths that undergo substantially less scattering in tissues [14,15]. This suggests that iRFP682 can overcome the limitations of GFP-like fluorescent proteins for imaging engrafted stem cells *in vivo*.

In our study, we labelled MSCs using an AAV-iRFP system, which enabled us to track labelled cells repeatedly until their death. We provided recombinant AAV (rAAV) with two independent expression cassettes, which ensured that the two proteins were expressed both independently and efficiently. In order to evaluate the practical feasibility of carrying out gene therapy for cancer, we added the TNF-related apoptosis inducing ligand (TRAIL) gene in another gene expression cassette. Many studies have demonstrated that TRAIL-induced apoptosis in tumour cells and TRAIL binding to its receptors on non-tumour cells does not commonly trigger apoptosis [16–19]. Thus, we believed that AAV-iRFP cell labelling technology represents a novel approach for monitoring MSC homing and survival *in vivo* and cell differentiation/apoptosis *ex vivo* by histological staining. These findings broaden the application of AAV-iRFP systems for MSC research and suggest that rAAV-iRFP cell labelling may be used for *in vivo* tracking of tumour cells after tumour formation.

## Materials and methods

### Recombinant vector construction

**Figure 1:**
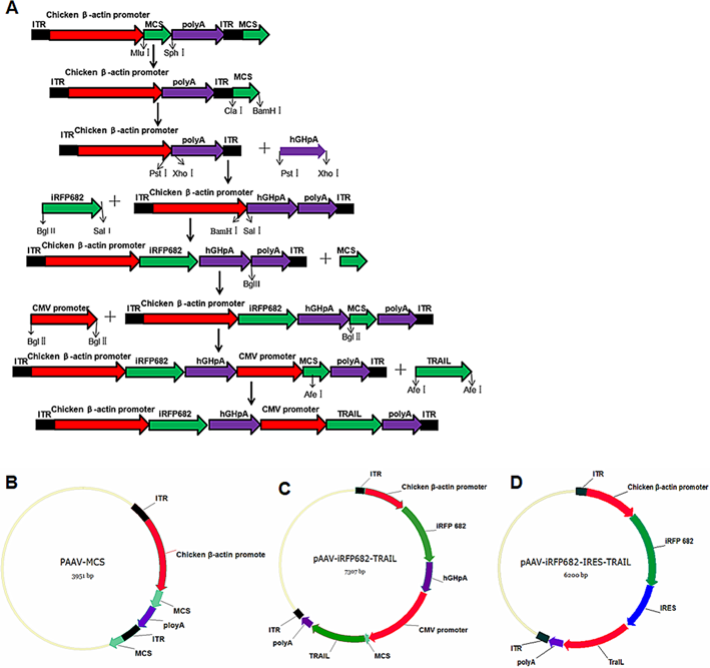
Schematic diagram of vector construction (**A**) Schematic diagram of recombination vector construction. (**B**) pAAV-MCS. (**C**) pAAV-iRFP682-TRAIL. (**D**) pAAV-iRFP682-internal ribosome entry site (IRES)-TRAIL.

pAAV-MCS, pSVAV2, and pDG plasmid vectors were constructed using standard molecular cloning methods and stored in our laboratory. First, we deleted previous double multiple cloning sites on the pAAV-MCS vector using a restriction endonuclease and a DNA polymerase. Subsequently, we amplified DNA products hGHpA, iRFP682, cytomegalovirus (CMV) promoter and TRAIL using PCRs, and obtained new MCSs using two complementary oligonucleotides annealed to become dsDNA (see Table 1). Gel fragments were extracted using a QIAquick Gel Extraction kit (OMEGA Bio-Tek, Doraville, U.S.A.). The purified DNA products were successively inserted into corresponding plasmid vectors. Finally, we obtained recombinant vector pAAV-iRFP682-TRAIL (see Figure 1A–C).

**Table 1:** Nucleotide sequences of primers used in the present study

Primer name	Sequences (5′–3′)	Restriction sites
hGHpA-F	GGAACCTGCAGGGATCCACGGGTGGCATCCCTGT	PstI, BamHI
hGHpA-R	GGAACCTCGAGAAGGACAGGGAAGGGAG	XhoI
iRFP-F	GGAACGTCGACGCCACCATGGCGGAAGGATCCGT	SalI
iRFP-R	GGAACAGATCTTCACTCTTCCATCACGC	BglII
MCS+	GATCTGTACACTAGTTTAAACCGGTCGACGGWCCGTACGGCGCGCCTAGGGCCCGGCCGGCC TTAAGCGCTAGCGATC	BsrGI, SpeI, PmeI, AgeI, RsrII, BsiwI, AscI, AvrII, ApaI, SfiI, FseI, AfeI, NheI, BmtI
MCS-	GATCGATCGCTAGCGCTTAAGGCCGGCCGGGCCCTAGGCGCGCCGTACGGWCCGTCGACC GGTTTAAACTAGTGTACA	BsrGI, SpeI, PmeI, AgeI, RsrII, BsiwI, AscI, AvrII, ApaI, SfiI, FseI, AfeI, NheI, BmtI
CMV promoter-F	GGAACAGATCTACGCGTGGAGCTAGTTAT	BglII
CMV promoter-R	GGAACAGATCTTCCCAATTCTTTGCCAAAGT	BglII
TRAIL-F	GGAACAGCGCTGCCACCATGGCTATGATGGAGGT	AfeI
TRAIL-R	GGAACAGATCTTTAGCCAACTAAAAAGG	AfeI

Another recombinant vector pAAV-iRFP682-IRES-TRAIL was obtained by deleting an expression cassette (including hGHpA and CMV-promoter) from pAAV-iRFP682-TRAIL and insert IRES between the two genes (Figure 1D). Each recombinant intermediate plasmid was transferred to the SURE-2-prepared competent cells and digested by restriction enzymes and sequencing (sequenced by Sangon Biotech Co. Ltd., Shanghai, China).

### Transfection of pAAV-iRFP682-TRAIL and pAAV-iRFP-IRES-TRAIL

The human embryonic kidney HEK-293 cell line was obtained from the cell bank of the Chinese Academy of Sciences (Shanghai, China). Monolayer cultures of HEK-293 cells were maintained in Dulbecco’s modified Eagle’s medium (DMEM) supplemented with 10% FBS and 1% penicillin and streptomycin. The cells were cultured on a 24-well plate at 37°C in a humidified incubator with 5% CO_2_. When they reached a confluence of approximately 70%, recombination plasmids pAAV-iRFP682-TRAIL and pAAV-iRFP682-IRES-TRAIL were used for cell transfection. Seventy-two hours later, infected cells were collected and then tested using a standard Western blot, as previously described by Ausubel et al. [20], to identify the expression of TRAIL. The blots were probed with rabbit anti-TRAIL (1:400; Boster, China) and horseradish peroxidase–conjugated goat anti-rabbit (1:10000; Abgent, U.S.A.) antibodies.

### Packaging, purification and identification of recombinant virus

According to the molar ratio of 1:1 (N/P =20), pAAV-iRFP682-MCS and packaging/helper plasmid pDG were separately co-infected into HEK-293 cells using the polyethylenimine transfection method, and the virus was then purified and concentrated using the CHCl_3_-PEG8000/NaCl-CHCl_3_ method. In addition, titres of recombinant virus solutions were measured by quantitative fluorescence PCR (QF-PCR) in an ABI 7500 Real-Time PCR system (Applied Biosystems, U.S.A.), and proteins were extracted using a QF-PCR kit (Tiangen Biotech Co. Ltd., Beijing, China). The QF-PCR analysis was carried out under the following conditions: 95°C for 3 min, 30 cycles at 94°C for 10 s and at 61°C for 40 s. The purity of recombinant virus solutions was measured using the Coomassie Blue staining method, and the genomes were extracted using a DNeasy blood and tissue kit (Cat. No. 69504; QIAGEN, Valencia, CA, U.S.A.).

### Recombinant virus transduction

MSCs were obtained from the cell bank of the Chinese Academy of Sciences (Shanghai, China) and cultured in six-well plates filled with 90% DMEM/F12 (Gibco, Grand Island, NY, U.S.A.) supplemented with 10% FBS (Gibco, Grand Island, NY, U.S.A.). Once the cells spread over 50% of the plate, the culture solution was removed and 500 μl DMEM/F12 was added to the plates. Afterwards, recombinant virus rAAV-iRFP682-TRAIL and helper virus rAAV-SVAV2 (stored in the laboratory) were added to the freshly applied aliquots of the medium. After 24 h of transduction in a humidified 5% CO_2_ incubator (Thermo Scientific BB150, U.S.A.) at 37°C, iRFP expression was observed and recorded under an inverted fluorescence microscope (ZEISS, Shanghai,China). When iRFP expression was stable after 72 h of transduction, total genomic DNA was isolated and integration into the AAVS1 site was detected using QF-PCR. The specific primer pairs used were AAV D-sequence sense primer HAIJP1 (5′-AGGAACCCCTAGTGATGGAG-3′) and human chromosome 19 AAVS1 site-specific primer HAIJP2 (5′-TCAGAGGACATCACGTGGTG-3′) [21], which were synthesized by Sangon Biotech Co. Ltd. (Shanghai, China). The programme was detailed as follows: 96°C for 3 min, 30 cycles at 98°C for 15 s and at 68°C for 2 min, as well as at 68°C for 5 min. The enzyme used to enhance PCR was KOD-Plus-Neo (Toyobo Life Science Co., Ltd., Tokyo, Japan). After PCR amplification, electrophoresis on 1% agarose gel was conducted to detect whether rAAV-iRFP682-TRAIL had been integrated into the AAVS1 locus on human chromosome 19.

### *In vivo* imaging

MSCs (1 × 10^6^ cells) in 100 μl PBS were injected into the fourth pair of mammary fat pads of nude mice. Imaging was performed 1 h after cell transplantation in anaesthetized mice using Kodak *In-Vivo* MultiSpectral Imaging System FX (2D; Kodak, Rochester, NY, U.S.A.) in epifluorescence mode equipped with 663/682 nm and 488/597 filters for excitation and emission, respectively. Images were taken with 60 s exposure. Signal intensity was represented by radiance and encoded by pseudocolours on the iRFP and enhanced GFP (EGFP) images. All animal experiments were performed in a facility approved by the Association for Assessment and Accreditation of Laboratory Animal Care.

### Statistical analysis

Transfection rate was determined by a flow cytometry analysis. Trail and β-actin relative protein expression levels were calculated using ImageJ software and the protein band to analyse the grey value. Statistical analysis was performed using GraphPad Prism 5.0 software (GraphPad Software, Inc., San Diego, CA). One-way repeated measure ANOVA and Student’s *t*test were used. Differences were considered signicant at *P<0.05 and **P<0.01.

## Results

### Vector construction and identification

**Figure 2:**
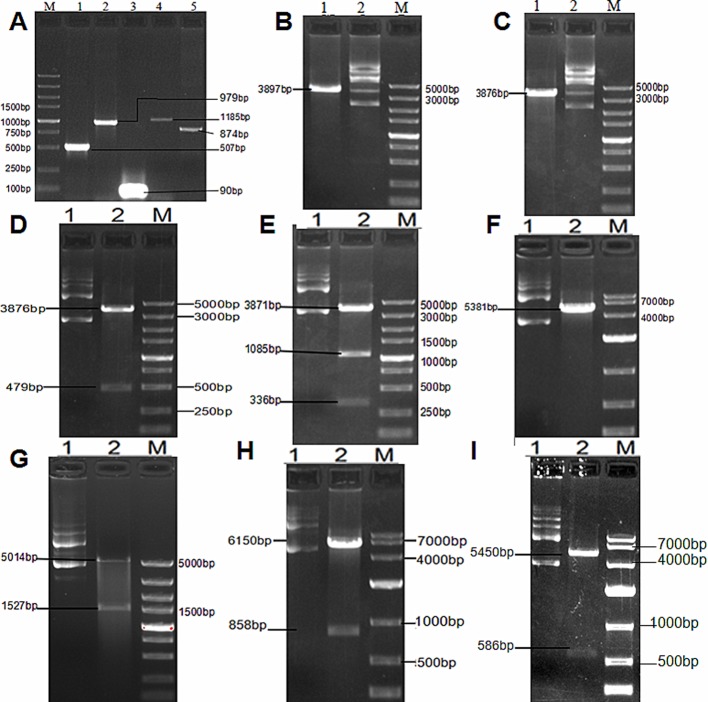
Verification of plasmid recombination products by agarose gel separation. (**A**) PCR amplification of hGHpA, iRFP682, MCS, CMV-promoter, and TRAIL. Lane M, DNA ladder; lane 1: PCR products of hGHPA; lane 2: PCR products of iRFP682; lane 3: PCR products of MCS; lane 4: PCR products of CMV-promoter; lane 5: PCR products of TRAIL. (**B**) pAAV-MCS lacking the first multiple cloning site verified by digestion. Lane 1: digested with EcoRI; lane 2: digested with SphI; lane 3: DNA ladder. (**C**) Plasmid lacking the second multiple cloning site verified by digestion. Lane 1: digested with BglII; lane 2: digested with BamHI; lane 3: DNA ladder. (**D**) Plasmid with double terminator verified by digestion. Lane 1: undigested; lane 2: digested with PstI and XhoI; lane 3: DNA ladder. (**E**) Plasmid with iRFP682 gene verified by digestion. Lane 1: undigested; lane 2: digested with SalI and XhoI; lane 3: DNA ladder. (**F**) Plasmid with new multiple cloning site verified by digestion. Lane 1: undigested; lane 2: digested with AfeI; lane 3: DNA ladder. (**G**) Plasmid with two complete expression cassettes verified by digestion. Lane 1: undigested; Lane 2: digested with BamHI and MluI; lane 3: DNA ladder. (**H**) Recombinant vector pAAV-iRFP682-TRAIL verified by digestion. Lane 1: undigested; lane 2: digested with AfeI; Lane 3: DNA ladder. (**I**) Recombinant vector pAAV-iRFP682-IRES-TRAIL verified by digestion. Lane 1: undigested; lane 2: digested with AscI and SphI; lane 3: DNA ladder.

hGHpA, iRFP682, MCS, CMV-promoter and TRAIL were amplified using PCR (see Figure 2A). After pAAV-MCS was digested by MluI/SphI and the self-ligation reaction dealt with, the produced plasmid vector lacked the first multiple cloning site (3897 bp) (see Figure 2B). Next, after pAAV-MCS was digested by ClaI/BamHI and the self-ligation reaction dealt with, the produced plasmid vector lacked the second multiple cloning site (3876 bp) (see Figure 2C). Subsequently, hGHpA was linked to the above plasmid vector digested by PstI/XhoI to obtain a recombinant vector with a double terminator (4355 bp) (see Figure 2 D). IRFP682 was linked to the previous plasmid vector digested by SalI/BamHI to obtain a recombinant vector with the iRFP682 gene (5292 bp) (Figure 2 E). A new second multiple cloning site was linked to the above plasmid vector digested by BglII to obtain a recombinant vector with a new multiple cloning site (5381 bp) (see Figure 2 F). CMV-promoter was linked to the previous plasmid vector digested by BglII to obtain recombinant vector pAAV-iRFP682-MCS with two complete expression cassettes (6541 bp) (see Figure 2G). TRAIL was linked to the above plasmid vector digested by AfeI to obtain recombinant vector pAAV-iRFP682-TRAIL (7303 bp) (see Figure 2H). The length between the two inverted terminal repeat sequences is 4.4 kb, which is not beyond the maximum packaging capacity of AAV (4.7 kb). IRES was linked to pAAV-iRFP682- TRAIL by deleting an expression cassette to obtain pAAV-iRFP682-IRES-TRAIL (6200 bp) (see Figure 2I).

### High expression of recombinant vector pAAV-iRFP682-TRAIL

**Figure 3:**
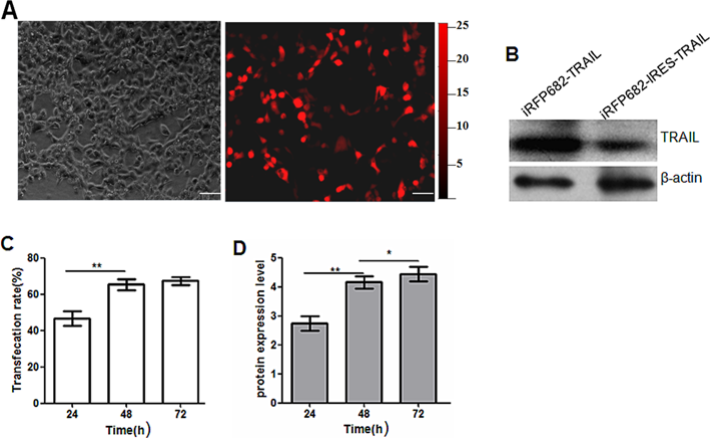
Transduction of HEK-293 cells with recombinant vectors (bars: 20 μm). (**A**) Expression of iRFP expression cassette in HEK-293 cells observed under a fluorescence microscope. (**B**) Western blot image of the TRAIL protein from two recombinant vectors. Lane 1: pAAV-iRFP682-TRAIL transfection; lane 2: pAAV-iRFP682-IRES-TRAIL transfection. (**C**) pAAV-iRFP682-TRAIL transfection rate after 24, 48 and 72 h. (**D**) TraiL protein expression levels at 24, 48 and 72 h.

In order to determine whether the two expression cassettes retained their basic functions, we transduced HEK-293 cells and, 24 h later, transfected cells showed robust and bright light under a fluorescence microscope (see Figure 3A). Western blot image revealed that the TRAIL protein from pAAV-iRFP682-TRAIL was expressed more highly than from the IRES-mediated recombinant vectors (see Figure 3B), which demonstrates that the expression of protein mediated by a complete expression cassette was productive. Transfection rate increased until 48 h and then remained stable (see Figure 3C). The changes of protein expression levels tended to follow the transfection rate (see Figure 3D).

### Recombinant vector pAAV-iRFP682-TRAIL can be well packed

**Figure 4:**
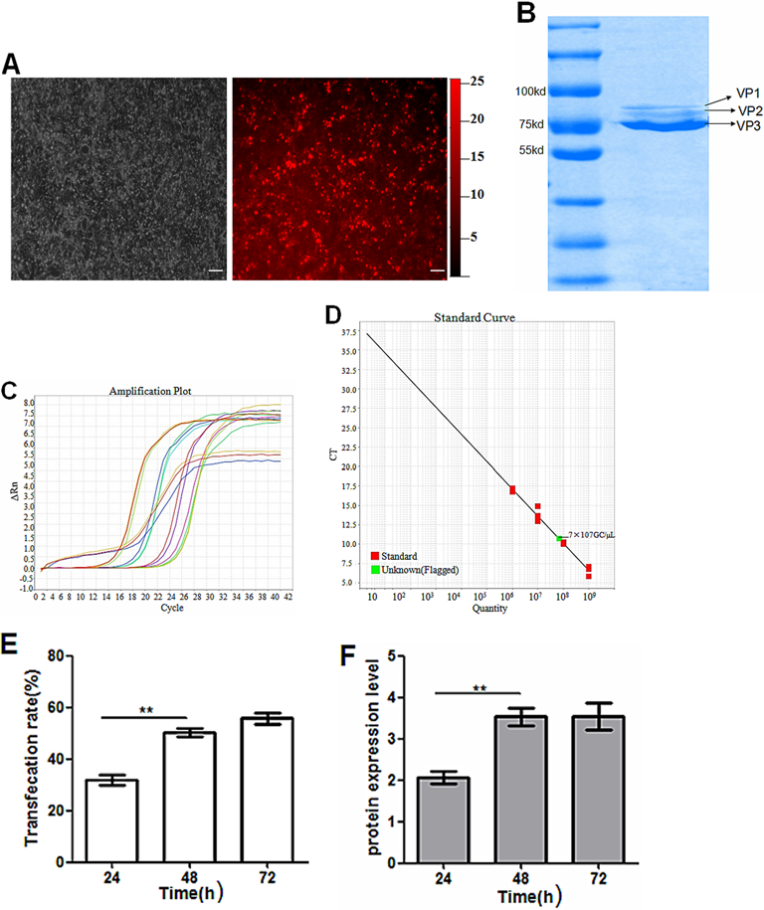
Packaging of viral vector rAAV-iRFP682-TRAIL (bars: 20 μm). (**A**) Fluorescence images of HEK-293 cells co-transfected with pAAV-iRFP682-TRAIL and pDG. (**B**) Purification detection by Coomassie Blue staining method; VP1, VP2, VP3 are the three capsid protein variants of AAV. (**C**) Amplification plot of TRAIL genes for plasmid standard specimens. (**D**) Standard curve for plasmids and quantification of viral specimens. (**E**) pAAV-iRFP682-TRAIL transfection rate after 24, 48 and 72 h. (**F**) TraiL protein expression levels at 24, 48 and 72 h.

pAAV-iRFP682-TRAIL and packaging  vector pDG co-transduced HEK-293 cells, most of the cells showed iRFP682 expression 48 h later (Figure 4A). There are three capsid protein variants of AAV: VP1, VP2 and VP3 with sizes 87, 73 and 61KDa respectively; the content radio of these proteins is 1:1:10. Coomassie Blue staining shows that our target band contained no hybrid proteins, which suggests that our recombinant virus rAAV-iRFP682-TRAIL sample was pure (Figure 4B). According to the QF-PCR amplification curve in Figure 4C, the amplification efficiency of the TRAIL gene is very high (99.9%). With reference to the standard curve, we calculated that the titre of recombinant virus rAAV-iRFP682-TRAIL reached 7 × 10^10^ GC/ml (see Figure 4D), which indicates that the level of the recombinant virus packaging is in the normal range. The transfection rate was not influenced by the packaging vector during the virus packaging process. Transfection rate increased until 48 h and then stabilized (see Figure 4E). The protein expression level followed the trend in the transfection rate (see Figure 4F).

### Recombinant virus successfully infected MSCs and integrated into AAVS1

**Figure 5:**
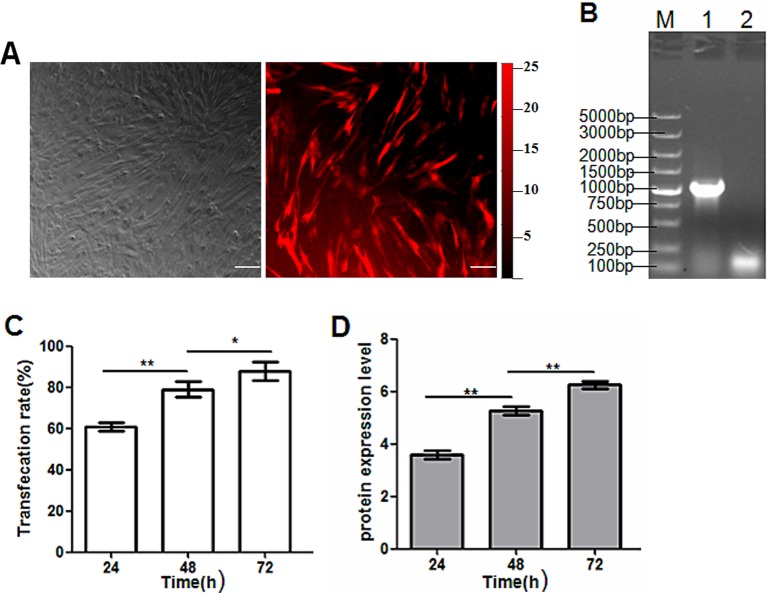
MSCs infected with recombinant AAV vectors. (**A**) Fluorescence microscopic image of iRFP expression in MSCs (bars: 20 μm). (**B**) Electrophoresis result of touchdown PCR products of DNA from infected MSCs. Lane M: DNA ladder; lane 1: touchdown PCR products of DNA from infected MSCs; lane 2: touchdown PCR products of DNA from non-infected MSCs. (**C**) pAAV-iRFP682-TRAIL transfection rate after 24, 48 and 72 h. (**D**) TraiL protein expression levels at 24, 48 and 72 h.

MSCs were labelled with iRFP after co-infection with recombinant and helper viruses. After 72 h, the expression of modified MSCs was both robust and stable, which proves that cell lines integrated with exogenous genes were successfully established (see Figure 5A). Following this, cells were collected and nuclear DNA extracted. Afterwards, touchdown PCRs were conducted and, only in the lane of the infected cells, a distinct PCR-amplified products band (1000 bp) was detected. However, this was not the case in the lane of the non-infected cells (see Figure 5B). This means that the integrants were located in the AAVS1 site in our experiment. The transfection rate increased almost constantly and reached the maximum at 72 h (see Figure 5C). The protein expression in modified MSC cells increased significantly (see Figure 5D).

### Mice can be labelled by MSCs modified with rAAV-iRFP682-TRAIL

**Figure 6:**
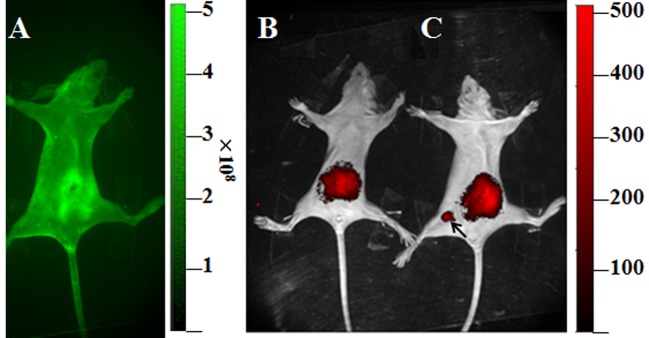
Expression of iRFP in living mice. (**A**) *In vivo* EGFP imaging after unmodified MSC injection. (**B**) *In vivo* iRFP imaging after unmodified MSC injection. (**C**) *In vivo* iRFP imaging after AAV-iRFP modified MSC injection.

Either unmodified or modified MSCs (10^6^ cells) were injected into female nude mice housed in sterile IVC cages; all animal work was approved by ethical review process and undertaken in-line with iRFP or EGFP signalling experimentation. Mice treated with either the modified MSC or the unmodified MSC showed a strong near-IR signal in the abdomen. However, strong near-IR signals were only observed in the fourth pair of mammary fat pads of nude mice treated with modified MSCs and no signals were detected in mice treated with unmodified MSCs in the same position. IR imaging indicated that iRFP fluorescence from MSCs can be readily detected after subcutaneous injection. On the other hand, iRFP682 with a high penetration depth in tissues had lower background interference compared with EGFP (see Figure 6).

## Discussion

rAAV is an increasingly popular gene transfer vector that has a number of advantages over others, including lack of pathogenicity and ability to mediate long-term gene expression in a variety of tissues *in vivo* and *in vitro*. AAV undergoes productive infection only in the presence of adenovirus or herpes virus. While in absence of helper viruses, AAV can establish latent infection in human cells through integration into a specific site (AAVS1) on the q arm of human chromosome 19 (19q13.4) [22,23]. The AAVS1 region seems to be a ‘safe harbour’ for gene delivery and transgenes integrated into the AAVS1 region demonstrate robust expression, which may last for several years. Our novel rAAV vector with two expression cassettes also showed good site-specific integration, avoiding risk from random chromosomal integration. The bottleneck that limits the large use of AAV is capacity. The capacity of our rAAV is approximately 4.7 kb, which led to high efficiency of virus packaging. Compared with the traditional AAV vector, rAAV with two expression cassettes demonstrated a more abundant protein yield.

Near-IR-based fluorescence imaging using iRFP gene labelling is novel technology with potential use for *in vivo* applications. Autoluminescence from tissues often interferes with the use of conventional GFP-like fluorescent proteins, including EGFP, DsRed and mCherry. However, when using near-IR fluorescence (NIRF) (from 650 to 900 nm), absorption of light by biological components such as haemoglobin, lipids and water is minimal, allowing light to reach its maximum depth of tissue penetration [24,25]. iRFP fluorescence labelling combines the advantages of luciferase for non-invasive, whole-animal imaging and GFP for cell imaging. At present, there are few studies on iRFP and the lack of an effective carrier is an important factor in restricting its development. The results of our research indicate that MSC labelled with iRFP is a good delivery vector to measure cell behaviour *in vivo*.

A previous study showed that *in vivo* images of iRFP-labelled solid tumours and iRFP-labelled cells cultured *in vitro* were well-observed [24]. However, the effect and distribution of spontaneous fluorescence after injecting iRFP-labelled cells *in vivo* remained unclear. Other studies revealed the characteristics of cells injected subcutaneously into the head and feet respectively and presented the figures of the head and feet on *in vivo* images [15,25]. In these positions, the interfering substances were positioned low in the body, which can lead to low spontaneous fluorescence. Thus, we are unaware of other parts of the body where iRFP could be detected. In the present study, we found that the whole organism except the abdomen had less spontaneous fluorescence under near-IR wavelengths, suggesting that iRFP-labelled cells could be used as an ideal real-time biomarker and for living cell tracking. However, the injection site and tracking route should not be near the abdomen.

In the present study, we constructed an expression vector containing two independent expression cassettes wrapped by AAV under the maximum packing capacity of the virus to ensure that TRAIL and iRFP were strongly overexpressed. We found that the new vector had good virus packaging characteristics and in site-specific integration. Our results also suggest that optical imaging can be used to study the homing mechanism and evaluate the therapeutic efficacy of engineered MSCs in real time. Furthermore, the findings indicate that the rAAV-iRFP system could provide a useful approach to track survival of engineered stem cells *in vivo*.

## Abbreviations

AAV: adeno-associated virus
ITR: inverted terminal repeat
VP: virus protein
AAVS1: adeno-associtated virus site 1
CMV: cytomegalovirus
DMEM: Dulbecco’s modified Eagle’s medium
F12: Ham’s F-12 nutrient mixture
HEK-293: human kidney cell line(-293)
pAAV: adeno-associated virus plasmid vector
MCS: multiple clone site
hGHpA: polyadensinic acid
N/P: nitrogen:phosphorus ratio
EGFP: enhanced GFP
FP: fluorescent protein
IRES: internal ribosome entry site
iRFP: IR fluorescent protein
MSC: mesenchymal stem cell
NIRF: Near-IR fluorescence
QF-PCR: quantitative fluorescence PCR
rAAV: recombinant adeno-associated virus
IVC: individually ventilated cages
TNF: tumor necrosis factor
TRAIL: TNF-related apoptosis inducing ligand
